# Nitric Oxide Down-Regulates Topoisomerase I and Induces Camptothecin Resistance in Human Breast MCF-7 Tumor Cells

**DOI:** 10.1371/journal.pone.0141897

**Published:** 2015-11-05

**Authors:** Nilesh K. Sharma, Ashutosh Kumar, Amrita Kumari, Erik J. Tokar, Michael P. Waalkes, Carl D. Bortner, Jason Williams, Marilyn Ehrenshaft, Ronald P. Mason, Birandra K. Sinha

**Affiliations:** 1 Immunity, Inflammation and Disease Laboratory, National Institute of Environmental Health Sciences, NIH, Research Triangle, Park, Durham, North Carolina, United States of America; 2 National Toxicology Program, National Institute of Environmental Health Sciences, NIH, Research Triangle, Park, Durham, North Carolina, United States of America; 3 Laboratory of Signal Transduction, National Institute of Environmental Health Sciences, NIH, Research Triangle, Park, Durham, North Carolina, United States of America; 4 Laboratory of Structural Biology, National Institute of Environmental Health Sciences, NIH, Research Triangle, Park, Durham, North Carolina, United States of America; Albany Medical College, UNITED STATES

## Abstract

Camptothecin (CPT), a topoisomerase I poison, is an important drug for the treatment of solid tumors in the clinic. Nitric oxide (^·^NO), a physiological signaling molecule, is involved in many cellular functions, including cell proliferation, survival and death. We have previously shown that ^·^NO plays a significant role in the detoxification of etoposide (VP-16), a topoisomerase II poison *in vitro* and in human melanoma cells. ^·^NO/^·^NO-derived species are reported to modulate activity of several important cellular proteins. As topoisomerases contain a number of free sulfhydryl groups which may be targets of ^·^NO/^·^NO-derived species, we have investigated the roles of ^·^NO/^·^NO-derived species in the stability and activity of topo I. Here we show that ^·^NO/^·^NO-derived species induces a significant down-regulation of topoisomerase I protein via the ubiquitin/26S proteasome pathway in human colon (HT-29) and breast (MCF-7) cancer cell lines. Importantly, ^·^NO treatment induced a significant resistance to CPT only in MCF-7 cells. This resistance to CPT did not result from loss of topoisomerase I activity as there were no differences in topoisomerase I-induced DNA cleavage *in vitro* or in tumor cells, but resulted from the stabilization/induction of bcl2 protein. This up-regulation of bcl2 protein in MCF-7 cells was wtp53 dependent as pifithrine-α, a small molecule inhibitor of wtp53 function, completely reversed CPT resistance, suggesting that wtp53 and bcl2 proteins played important roles in CPT resistance. Because tumors *in vivo* are heterogeneous and contaminated by infiltrating macrophages, ^·^NO-induced down-regulation of topoisomerase I protein combined with bcl2 protein stabilization could render certain tumors highly resistant to CPT and drugs derived from it in the clinic.

## Introduction

Nitric oxide (^·^NO) is generated intracellularly from L-arginine by nitric oxide synthase (NOS). Three forms of NOS have been identified, including neuronal (nNOS), endothelial (eNOS) and a Ca^2^+-independent inducible isoform of NOS (iNOS) [1, 2]. ^·^NO is now recognized to have a biphasic effects on cells: at low concentrations (<50 nM), it acts as a tumor promoter while at higher concentrations (>300 nM), it causes DNA damage and cell death [3–6]. ^·^NO modulates hypoxia inducible factor (HIF 1), prolyl hydroxylase (PHD2) enzyme, and the bcl2 family of proteins [7–11]. This modulation may involve nitrosation of sulfhydryl groups, resulting in modification of the activity, stability and regulation of protein expression via posttranslational modifications that induce inhibition of proteasomal degradation [8, 11]

Topoisomerases constitute an important class of nuclear enzymes responsible for maintaining the topology of DNA and are involved in DNA repair, transcription, replication and segregation of chromosomes [12–16]. Inhibition and/or interference with topoisomerase functions lead to cell death [13–17]. Several clinically active anticancer drugs (e.g., Etoposide, Adriamycin, and Camptothecin) target topoisomerases [13, 14, 16]. Camptothecin (CPT), a topoisomerase I (topo I) poison, is effective against a wide variety of solid tumors. CPT stabilizes transient complexes formed between topo I and DNA (cleavable complexes) resulting in the formation of highly toxic double strand breaks. CPT cytotoxicity also depends upon many other variables, including cellular topo I levels and the ability of cells to repair DNA damage and undergo apoptosis [13, 18–21]. An important determinant of the sensitivity of CPT is the presence of functional p53 protein (wtp53) and its ability to appropriately respond to DNA damage, repair, and commitment to undergo apoptosis [12, 22, 23].

Both topo I and II contain several cysteines, and modification of free SH groups in topo II has been shown to decrease the catalytic activity of the protein [24]. Because tumors are heterogeneous and contaminated with infiltrating macrophages, ^·^NO is continuously generated [25, 26]. Furthermore, during inflammation large amounts of ^·^NO are formed that can diffuse into tumor tissue/cells and affect the stability and/or activity of proteins. S-nitrosylation (or nitrosation) is increasingly recognized as important in biologically signaling /regulatory mechanisms involving protein free SH groups [27, 28]. Because topo I and topo II contain a number of reactive SH groups, reactions with intracellularly generated ^·^NO/^·^NO-derived species in tumors could result in modification of the activity/stability of these cellular proteins, and thus, may compromise treatment of patients with topo-active drugs. In this regard, we have shown that ^·^NO/^·^NO-derived species directly reacted with the anticancer drug VP-16 and rendered it inactive against tumor cells [29, 30].

In this article we show, for the first time, that topo I is nitrosated by ^·^NO/^·^NO-derived species *in vitro* and is significantly down-regulated in both the HT-29 colon and MCF-7 breast cancer cell lines. We choose these cell lines because they contain high levels of topo I [20, 31, 32] and mutant p53 and wild type p53, respectively. We also examined the cytotoxicity of CPT, whose cellular pharmacology is well characterized [20, 22] in these two cell lines, following treatment with PPNO, an ^·^NO donor. We found that reaction of ^·^NO/^·^NO-derived species (generated chemically or intracellularly) with free SH group of topo I protein (nitrosation of topo I) induces significant down-regulation of topo I protein in both cells by a proteasomal degradation pathway. However, the toxicity of CPT was only significantly affected in MCF-7 cells, indicating that wtp53 plays an important role in tumor cell killing by CPT.

## Materials and Methods

Camptothecin was a gift of the Drug Synthesis and Chemistry Branch, Developmental Therapeutic Program of NCI, NIH. Human topo I, antibody to human topo I, Supercoiled pHOT1 DNA, and SDS/KCl precipitation assay kits were obtained from Topogen (Port Orange, FL). The nitric oxide donor, propylamine propylamine nonoate (PPNO), was obtained from Cayman Chemicals (Ann Arbor, MI). A stock solution of PPNO was prepared in 0.2 N NaOH and was stored at -80^°^C. Pifithrin-α, cysteine, sodium nitrite, ammonium sulfamate, mercuric chloride, sulfanilamide and N-1-naphthylethylenediamine dihydrochloride were obtained from Sigma-Aldrich Chemical Company (St. Louis, MO). Primary antibodies for the analysis of bcl2, Bax, p53, and p21 were from Santa Cruz Biochemicals (Dallas, TX). A nitric oxide assay kit was purchased from Thermo Scientific (Waltham, MA). Anti-s-nitroso-cys (SNO-Cys) was purchased from Alpha Diagnostic International, San Antonio, TX, USA)

### Confocal microscopy for nitrosation of topo I in cells

About 1 X 10^5^ cells were plated for 18 h for at 37°C in culture plates on glass coverslips. The media was removed and replaced with fresh media and treated with 100 μM PPNO for 18 h. To investigate the extent of nitrosylation and co-localization of s-nitrosated adducts with topo I following PPNO treatment, the cells were fixed with 4% paraformaldehyde for 15 minutes at room temperature, washed twice for 5 minutes, permeablised for 5 minutes with 0.5% Triton X-100, and washed twice for 5 minutes. After blocking with 4% fish gelatin in PBS (pH 7.4) for 2 h at room temperature, the cells were incubated with rabbit anti-s-nitrosylated-cysteine igg (diluted 1:2000) and mouse anti-topo I (diluted 1:2000) for 2 h, followed by secondary anti-rabbit Alexa Fluor 488 and anti-mouse Alexa Fluor 568 antisera (both diluted 1:1000) for 1 h. Coverslips containing cells were washed four times and mounted on glass slides using Prolong Gold anti-fade reagent. Confocal images were taken with a Zeiss LSM 510-UV Meta microscope (Carl Zeiss Inc., Oberkochen, Germany) using a Plan-NeoFluar 40X/1.3 Oil DIC objective with zoom 3. The 488 nm line from an Argon laser was used for producing polarized light for fluorescence excitation of the Alexa Fluor 488 secondary antibody. All images were acquired with equal excitation power (5%) and identical detection gain (532 volts).

### Cell culture

Human breast MCF-7 and colon cancer HT-29 cell lines (ATCC, Rockville, MD) were grown in Phenol Red-free RPMI media supplemented with 10% fetal bovine serum and antibiotics. MCF-7 cells and HT-29 cells were routinely used for 25 passages, after which the cells were discarded and a new cell culture was started from fresh frozen stock. For the treatment with PPNO (25–100 μM), cells were plated in RPMI media containing 0.5–1.0% FBS without any antibiotics. After the prescribed treatment period (0–18 h), the medium was changed to a regular medium containing 10% FBS and antibiotics.

### Cytotoxicity studies

The cytotoxicity studies were carried out by a cell growth inhibition assay. For the cell count-based growth inhibition studies, 150,000–250,000 cells/well were plated in 2 ml of complete medium onto a 6-well plate (in triplicates) and allowed to attach for 18 h. The medium was removed and fresh warm medium, containing 0.5–1% FBS without antibiotics and 100 μL of PBS (pH 7.0), was added. Cells were treated with PPNO (25–100 μM) for 6 h, followed by the addition of various concentrations of CPT, and cells were incubated for 48 h in the complete medium. DMSO was included as the vehicle control. For the cytotoxicity studies with PFT-α, cells were preincubated with PFT-α first for 1h before CPT or PPNO was added and incubated for an additional 48 hrs. Cells were trypsinized and the numbers of surviving cells were determined by counting the cells in a cell counter (Beckman, Brea, CA). We used PPNO as our ^·^NO donor as it has a short half-life (15 minutes at 37^°^C, pH 7.0), and found PPNO to be the least toxic to cells compared to various other ^·^NO-donors (e.g., Diproylenetriamine Nonoate). Even at 100 μM, only 10–15% of cells were found to be dead after 48 h of PPNO exposure.

### SDS-KCl precipitation assay

The formation of covalent topo I/DNA complexes with CPT under various conditions with HT-29 and MCF-7 cells was quantitated by the SDS-KCl precipitation assay as described by Liu et al. [33]. Briefly, DNA of cells growing in the logarithmic phase (2–3 x 10^6^ /ml) was labeled with [methyl-^3^H]-thymidine (10 μci, 20Ci/mmol; Perkin-Elmer, Waltham, MA) for 18–24 h. Cells were collected and washed twice with the medium, diluted in fresh medium and seeded into a six-well plate at a density of 1 x 10^5^ cells/ml. CPT, dissolved in DMSO, was added and incubated for 1 h. Cells were washed with PBS (2 x), and lysed with 1 ml of prewarmed lysis solution (Topogen). After lysis and shearing of DNA, DNA-CPT-topo I-complexes were precipitated with KCl. The precipitate was collected by centrifugation, and washed extensively (4 x) with the washing solution (Topogen) according to the manufacturer’s instructions. The radioactivity was counted in a scintillation counter after adding 5 ml of scintillation fluid. PPNO alone had no significant effect on SDS-KCl precipitate formation.

HT-29 cells were induced as previously described [34]. Briefly, HT-29 cells (5 x 10^6^ cells/T-75 ml flask) were treated with IL-1β (10 ng/ml), TNFα (10 ng/ml) and INFγ (10 ng/ml) for 18 h. The medium was removed and cells were washed once with the complete medium (RPMI 1640 containing 10% FBS and antibiotic mixture) before carrying out cytotoxicity studies. For the SDS-KCL precipitation studies with the induced cells, cells were prelabeled with ^3^H-Thymidine for 6–8 h before carrying out the induction as described above. However, ^3^H-thymidine was also present during the induction phase.

### Western blot assay

Samples (10 μg of total protein) were electrophoresed under reducing conditions through 4–12% Bis-Tris NuPage acrylamide gels (Invitrogen, Carlsbad, CA). After electrophoresis, proteins were transferred onto a nitrocellulose membrane and probed with anti-topo I, -p53, -p21, -bcl2, -Bax and -beta actin antibodies. An Odssey infrared imaging system (Li-Cor Biosciences, Linas coln, NE) was used to acquire images.

### Real-time reverse transcription–polymerase chain reaction

Gene expression levels in both cell lines were measured by RT-PCR analysis and were carried out as described previously [35]. Total RNA was isolated from cells using TRIzol reagent (Invitrogen, Carlsbad, CA) and purified with RNeasy mini kit columns (Qiagen, Valencia, CA) according to protocols from manufacturers. Purified RNA was reverse transcribed to cDNA with the use of MuLV reverse transcriptase (Applied Biosystems, Foster City, CA) and oligo-dT primers. Primers were designed with the use of Primer Express 3.0 software (bcl2, Bax; Applied Biosystems).

### Flow cytometry for apoptosis studies

#### Caspase Activity

Caspase activity for caspase-3/7-like enzymes was measured using a CaspaTag *in situ* assay kit (Chemicon, Billerica, MA) according to the manufacturer’s instructions. Briefly, 1 h prior to analysis, 300 μl of cells was added to 10 μl of a 30X CaspaTag reagent stock and incubation was continued. After the 1-h incubation, the cells were washed in 2 ml of CaspaTag wash buffer and then suspended in 500 μl of 1X PBS. Immediately prior to flow cytometric analysis, propidium iodide (PI) was added to a final concentration of 5 μg/ml. Cells were analyzed using an LSRII flow cytometer (Benton Dickinson, San Jose, CA) equipped with FACSDiVa software. CaspaTag and PI were excited using a 488 nm and 561 nm lasers and detected using a 530/30 nm and 582/15 nm filter, respectively. For each sample, 10, 000 cells were analyzed using FACSDiVa software.

#### Annexin-V binding assay

Changes in membrane phosphatidylserine symmetry were determined using an Annexin-5 assay kit (Trevigen, Gaithersburg, MD) according to the manufacturer’s instructions. Briefly, cells were washed in 1X PBS, then incubated with 2 μl Annexin-V FITC and propidium iodide (PI) in Annexin-V binding buffer for 15 minutes at room temperature. After this time, the samples were diluted with binding buffer and examined immediately by flow cytometry. Cells were analyzed using an LSRII flow cytometer (Benton Dickinson) equipped with FACSDiVa software. Annexin-V FITC and PI were excited using a 488 nm and 561 nm laser and detected using a 530/30 nm and 582/15 nm filters, respectively. For each sample, 10,000 cells were analyzed using FACSDiVa software.

### Spectrophotometric analysis for nitrosation of topo I

The number of reactive SH groups that could be modified by ^·^NO/ NO-derived species in topo I protein were estimated by a colorimetric assay using the diazotization reaction of sulfanilamide and coupling with N-1-naphthylethylenediamine as described previously [36, 37]. Cysteine was used as the standard. Topo I used for these studies was obtained from ProSpec Biotech Co (East Brunswick, NJ). It did not contain BSA or DTT as preservatives.

### Carbamidomethylation and mass spectrometry

The availability of free SH groups that could be modified in topo I by PPNO treatment was also confirmed by mass spectrometric analysis as described by Hasinoff et al. (2005). Briefly, 2 μg human topoI dissolved in 50 mM ammonium bicarbonate (pH 7.8) were incubated in the presence of freshly made 10 mM iodoacetamide for 30 minutes in the dark at 25^°^C. The reaction was quenched by the addition of freshly made 30 mM dithiothreitol and incubated at 25^°^C for 15 minutes. 2 μg of carbamidomethylated or untreated human topo I were digested with 20 ng of trypsin (Promega) at 37 oC for 4 hours. NanoLC-ESI-MS and MS/MS and MALDI-MS and MS/MS analyses were then performed using either an Agilent 1100 nanoLC system on-line with an Agilent XCT Ultra ion trap mass spectrometer with the Chip Cube Interface or an Applied Biosystems 4800 ToF/ToF mass spectrometer.

For NanoLC-ESI experiments, 20 μL of the peptide mixture from the tryptic digest (approximately 10 picomoles) were loaded onto an Agilent C_18_ chip (75 mm x 43 mm) followed by a 15 minute wash of 5% acetonitrile, 0.1% formic acid. Peptides were eluted by applying a linear gradient from 5% acetonitrile, 0.1% formic acid to 50% acetonitrile, 0.1% formic acid to the column over 45 minutes. This was followed by a 5 minute gradient from 50% acetonitrile, 0.1% formic acid to 95% acetonitrile, 0.1% formic acid and then a 10 minute hold at 95% acetonitrile, 0.1% formic acid. The mass spectrometer was used in the positive ion, standard enhanced mode and included settings of a mass range from 200 to 2200 m/z, an ionization potential of 2.1 kV, an ICC smart target of 200000 or 200 milliseconds of accumulation, and a 1.0 volt fragmentation amplitude. MS/MS data were acquired using a data dependent acquisition format with the 6 most abundant ions from each MS scan further interrogated by MS/MS. The automated switching for MS/MS required a threshold of 10000 counts. A peak list was generated from the data obtained from the nanoLC-ESI-MS/MS analysis using the Data Extractor feature of the Spectrum Mill software from Agilent. The Data Extractor settings included limiting the data search to deconvolved ions observed between 400 and 5000 Da and a retention time between 10 minutes and 50 minutes. MS scans with the same precursor mass (+/- 1.5 m/z) and retention time within 30 seconds were merged. Moreover, of the remaining MS/MS spectra, only spectra that contained sequence tag information greater than 2 residues were submitted for database searching. The resulting extracted data were then searched against the SwissProt/UniProt human and rodent databases using the MS/MS Search function in the Spectrum Mill software. Search settings included a trypsin specificity with one missed cleavage allowed, a precursor ion mass tolerance of 3 Da, a product ion mass tolerance of 0.7 Da, variable methionine oxidation and cysteine carbamidomethylation allowed, and a minimum matched spectral intensity of 70%.

For MALDI-MS experiments 0.4 mL of the topo I tryptic digests were spotted onto a stainless steel target where it was mixed with 0.33% saturated solution of alpha-cyano-4-hydroxycinnamic acid in 50:50 0.2% formic acid:acetonitrile. The instrument was employed in the positive ion and reflector modes for both the MS and MS/MS experiments and was calibrated for MS externally with a peptide calibration mixture (Applied Biosystems) and for MS/MS externally with the fragments of angiotensin I. Data were processed and searched using the Mascot search engine (Matrix Science) via the GPS Explorer interface (Applied Biosystems). The search was conducted using the SwissProt/UniProt database and included search settings of up to two missed tryptic cleavages, a precursor ion mass tolerance of 0.2 Da, and a product ion mass tolerance of 0.2 Da, variable methionine oxidation and cysteine carbamidomethylation were allowed.

### Statistical analysis

All experiments were repeated at least 3 times (n = 3) in triplicate. One-way analysis of variance (ANOVA) was used for statistical analysis. The Newman-Keuls post-test was used for multiple comparisons. The results are expressed as mean ±SEM. The differences were considered statistically significant when p values were less than 0.05.

## Results

### Assay for nitrosation of topo I

Reaction of ^·^NO with topo I protein was examined by spectroscopic analysis following treatment with excess acidified NaNO_2_ and diazotization reactions. Our studies indicate that topo I was nitrosated and resulted in the reaction of 4.5 ± 0.5 equivalents of free SH groups/mole of the protein. This suggests that there are between 4–5 free cysteines capable of reacting with ^·^NO/^·^NO-derived species in topo I protein; this was further confirmed using mass spectrometry.

### Mass spectrometric analysis

“Bottom up” mass spectrometric analyses were performed on tryptic digests of carbamidomethylated and untreated human topo I (S1 Table and S1 Fig). In the case of untreated topo I, these analyses resulted in the assignment of 19 MS and MS/MS spectra to 16 distinct tryptic peptides from topo I. These peptides, however, only covered approximately 23% of the topo I sequence, although a large portion of the lysine-rich N-terminus, amino acid residues 1–247, was not expected to be observed. Fortunately, even though only a modest percentage of the total protein sequence was observed, 5 of the 8 cysteine residues (C341, C386, C504, C505, and C733) in human topo I were observed. All 5 cysteines were observed to be unmodified under standard conditions with the caveat that any low stoichiometry modification may have gone undetected. Furthermore, it is unclear whether C504 and C505 were observed as a reduced or disulfide bonded species. The average mass error across all observed peptides was 0.4 Da, but the mass error for tryptic peptide 126 that contains residues C504 and C505 had a mass error of -0.90 Da. This mass error could be result of the relatively low mass accuracy of the ion trap instrument used for data acquisition or it could be reflective of the 2 Da mass loss that occurs during disulfide bond formation. It must be noted, however, that even if the residues C504 and C505 were observed as a disulfide bonded form of the peptide, this result does not necessarily reflect the red-ox state of the cysteines *in vivo*, as cysteine containing peptides can rapidly form disulfide bonds on the benchtop.

The analyses of carbamidomethylated topo I resulted in the identification of 30 MS and MS/MS spectra matching to 25 unique peptides, but still only 38% sequence coverage. Again, even though relatively low sequence coverage was observed, 5 of the 8 cysteine residues were observed (C300, C386, C504, C505, and C630), but only 3 (C386, C504, and C505) were in common with the untreated TOP1 samples. Importantly, the carbamidomethylation reaction was conducted at moderate iodoacetamide concentrations and mild conditions (buffered solution, 25^°^C, no added reductants, detergents or chaotropes) in efforts to only carbamidomethylate solvent-exposed and reactive cysteines. In fact, C504, C505, and C630 were all observed only in their carbamidomethylated form and C300 was observed as both the unmodified and carbamidomethylated species, suggesting that these residues can rapidly react with the aqueous iodoacetamide. C386, however, was observed only in the reduced sulfhydryl form, suggesting that this residue is protected and not able to react with iodoacetamide in the native protein.

### Confocal microscopy for topo I nitrosation in HT-29 and MCF 7 cells

We also examined nitrosation of topo I in these tumor cell lines. We found that treatment of either MCF-7 or HT-29 with 100 μM PPNO resulted in significant increase in nitrosation of a number of proteins in these cell lines and as indicated by co-localization experiments, some nitrosation of topo I was also evident in both cells (S2 Fig; yellow color, indicated by arrow). Interestingly, more nitrosation of topo I was observed in MCF-7 cells than HT-29 cells. However, it is not clear that this small difference observed in nitrosation of topo I between these cells lines played any role in significant CPT resistance observed in MCF-7 cells.

### Effects of ^·^NO on topo I protein

**Fig 1:**
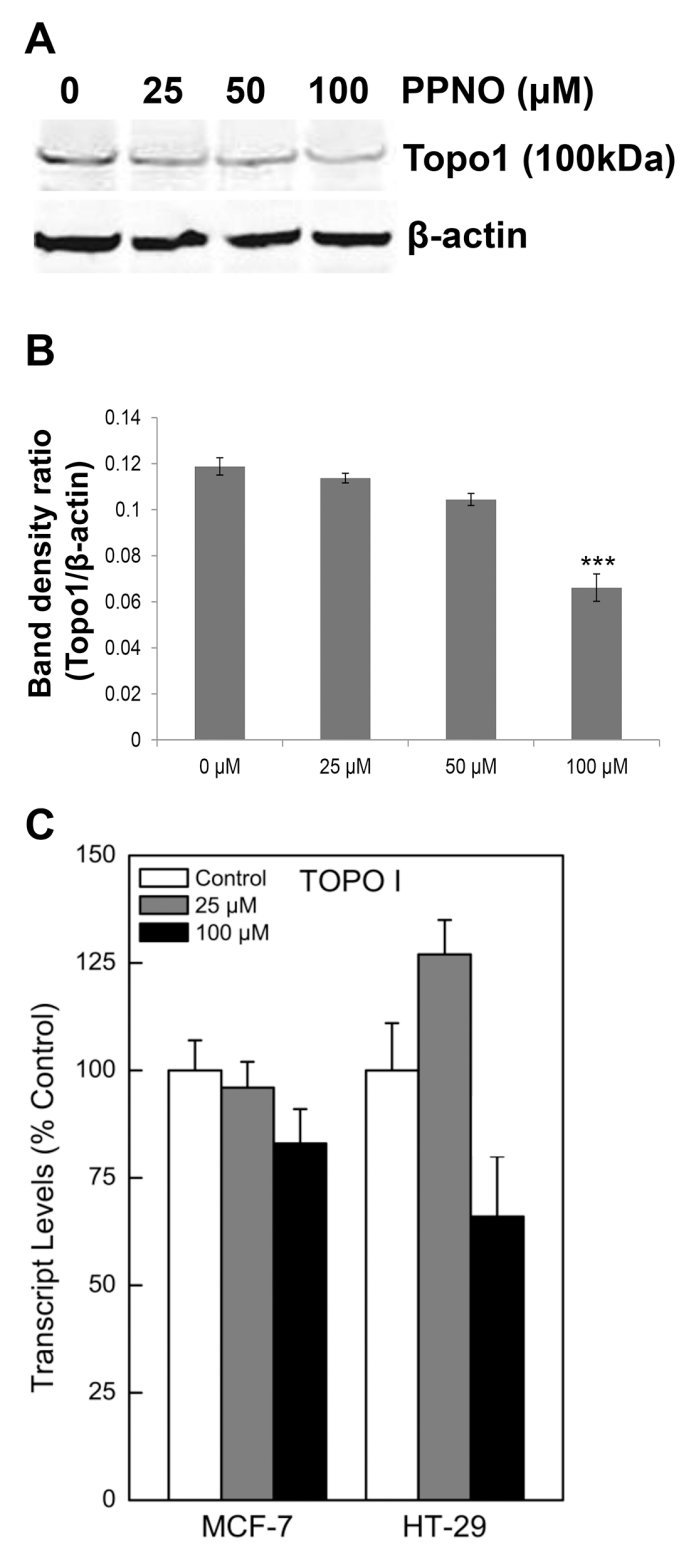
^·^NO-induced down-regulation of topo I protein levels in HT-29 cells. **(A)** HT-29 cells were treated with various concentrations of PPNO for 6 h as described in the Methods section. Cells were collected, lysed and analyzed by Western blots for topo I. **(B)** Quantification of topo I protein levels relative to actin, and **(C)** Transcript levels of the topo I gene in HT-29 and MCF-7 cells. Transcript levels were determined as described in the Methods section using actin as the control.

**Fig 2:**
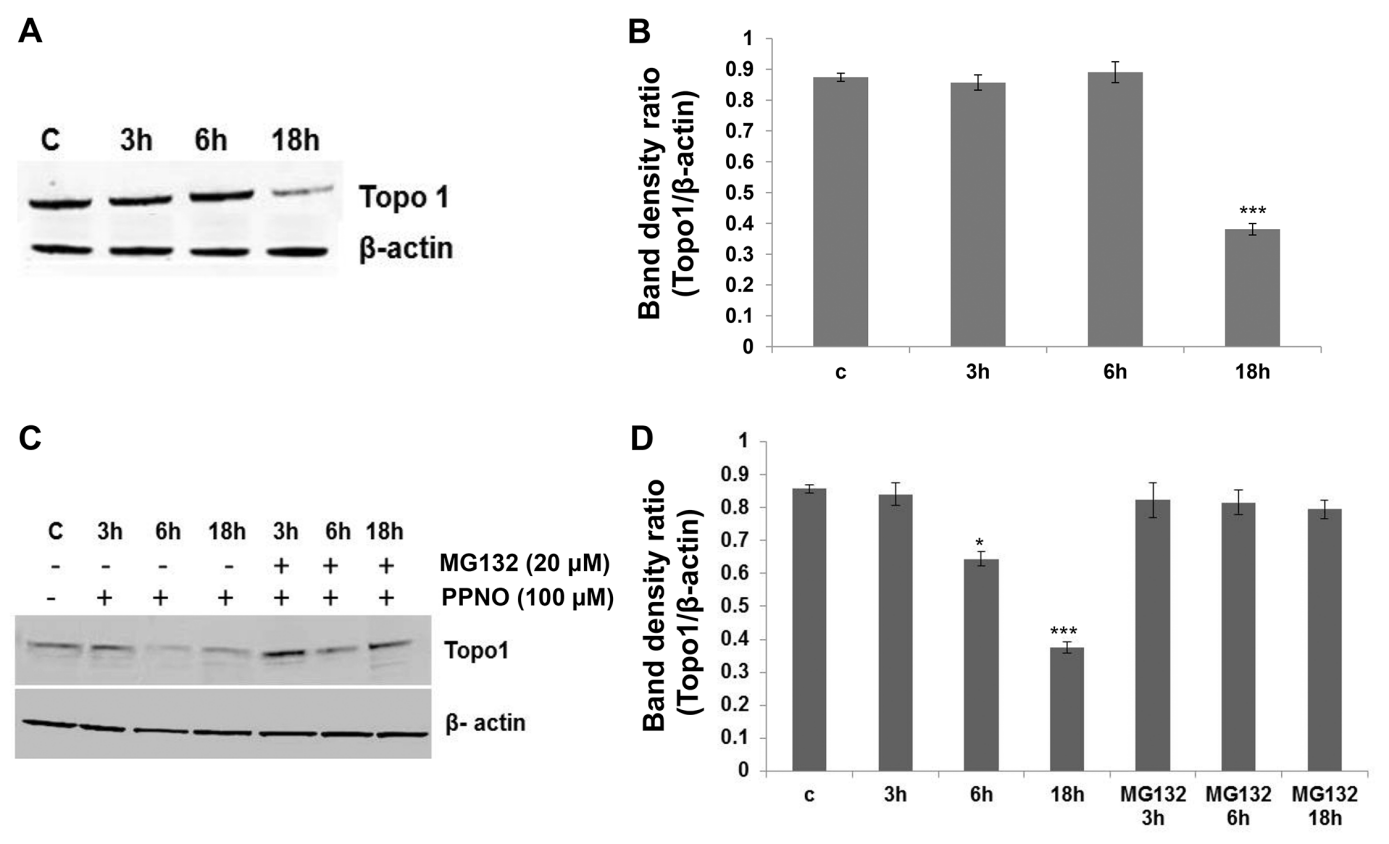
^·^NO-induced down-regulation of topo I protein levels in induced HT-29 and control HT-29 cells and effects of MG132. **(A)** HT-29 cells were induced with a cytokine mixture as described in the Methods section and analyzed for topo I protein by Western blot, **(B)** Quantification of the topo I protein, **(C)** Effects of MG132 on topo I levels following PPNO (100 μM) treatment with time, and **(D)** Quantification of the topo I protein levels.

While free SH groups do not directly react with ^·^NO, ^·^NO-derived species are known to nitrosates SH groups [27]. Therefore, we examined the effects of ^·^NO/^·^NO-derived species on the SH groups of topo I protein. Treatment of HT-29 cells with PPNO resulted in a dose- and time-dependent decrease in the levels of topo I protein, and a significant decrease in the protein levels was observed following 6 h of treatment with 100 μM PPNO (Fig 1). To further confirm that this decrease in topo I levels resulted from reaction with ^·^NO/^·^NO-derived species, we induced iNOS in HT-29 cells with a cytokine mixture (IL-β, TNFα and IFN-γ) as described previously [34]. Under these conditions, iNOS is rapidly induced, resulting in significant formation of ^·^NO. Under our experimental conditions and using Greiss reagent, the nitrite concentration was found to be 4.1± 0.8 μ*M*/10^7^ cells after 18 h of induction. In control cells and at 6 h of induction, the nitrite was not detectable. When cells were induced and the levels of topo I were analyzed by Western blot, a significant decrease in the topo I protein level was observed only after 18 h (Fig 2A and 2B), suggesting that the decrease in the protein levels in HT-29 cells was due to the formation of ^·^NO/^·^NO-derived species.

Because ^·^NO is known to induce posttranslational modifications, we evaluated both the effect of ^·^NO (via PPNO) on topo I mRNA and the proteasomal degradation of topo I protein. PPNO had no significant effect on the mRNA of the topo I gene (Fig 1C), suggesting that ^·^NO does not affect topo I gene regulation. However, MG132, an inhibitor of the ubiquitin/26 S proteasome, inhibited topo I protein degradation (Fig 2C and 2D), suggesting that the reacted/nitrosated topo I protein is degraded via a proteasomal pathway.

### Cytotoxicity studies in HT-29 cells

**Fig 3:**
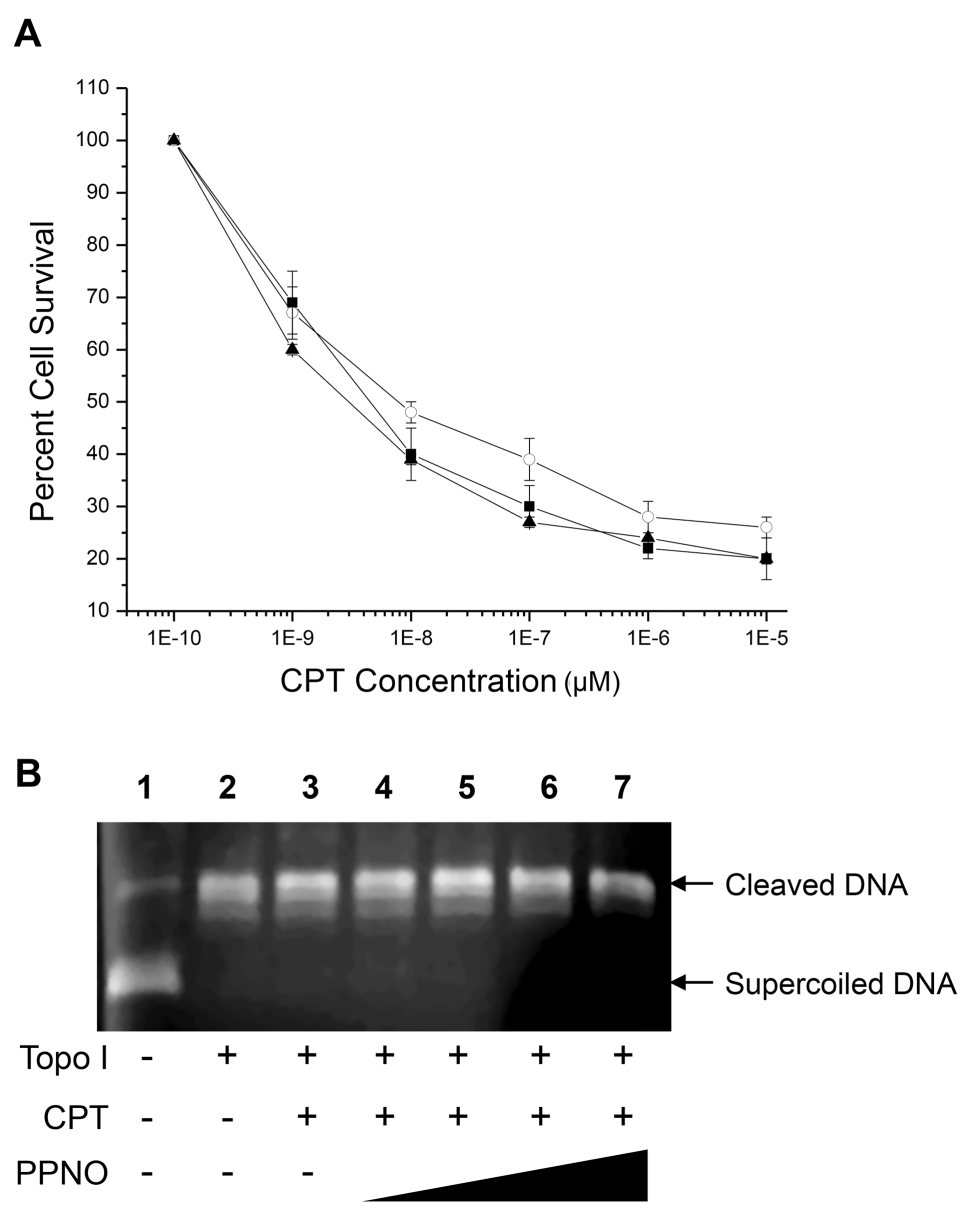
Cytotoxicity of CPT in HT-29 cells. **(A)** Cell count-based cytotoxicity of CPT was carried out as described in the methods sections. HT-29 cells were seeded in a 6-well plate in triplicates and allowed to attach for 18 h. PPNO (100 μM) treatment was carried out in medium containing 0.5–1% FBS without antibiotics for 6 h. Medium was changed to 10% FBS before adding various concentrations of CPT, and incubated for 48 h, and surviving cells were counted. Data represent at least three separate experiments. CPT alone (■-■); CPT in the presence of PPNO (○-○) and cytokine-induced HT-29 cells (▲-▲). **(B)** DNA cleavage induced in pHOT1 DNA by CPT (10 μM) in the presence of topo I (5 units) and various concentrations of PPNO. The DNA cleavage assay was carried out as described in the Methods section. Lane 1, control DNA; lane 2, in the presence of topo I; lane 3, in the presence of 10 μM CPT and topo I; lanes 4–7, in the presence of 25, 50, 100 and 200 μM PPNO, 10 μM CPT and topo I, respectively.

We evaluated whether an ^·^NO-mediated degradation of topo I protein results in altered sensitivity to CPT. PPNO had no significant effects on CPT cytotoxicity in HT-29 cells (Fig 3A), suggesting that down-regulation of the topo I protein level in HT-29 cells does not contribute significantly to CPT toxicity. Similar results were observed in HT-29 cells induced by a cytokine mixture (Fig 3 **;** Table 1).

**Table 1:** Cytotoxicity (IC_50_, μM) of CPT and cleavable complex formation in the presence or absence of PPNO.

		Cleavable complexes formed^#^		
	Cytotoxicity	CPT Concentration		
Cell line	(IC_50,_ μM)	1μM	5 μM	25 μM
HT-29	0.005 ± 0.001	3.7 ± 0.4	3.7 ± 0.5	5.7 ± 1.0
HT-29 + PPNO	0.007 ± 0.001	3.4 ± 0.6	5.0 ± 1.0	6.1 ± 1.0
HT-29, Induced	0.004 ± 0.001	2.1 ± 0.2	4.0 ± 0.3	6.3 ± 0.3
MCF-7	0.15 ± 0.04	2.0 ± 0.4	4.2 ± 1.0	5.2 ± 0.5
MCF-7 + PPNO	2.0 ± 0.05 ***	2.0 ± 0.4	3.6 ± 0.5	4.2 ± 0.5

Cytotoxicity studies were carried out as described in the Methods section. The concentration of PPNO was 100 μM, and cells were pretreated with PPNO for 6 h before the various concentrations of CPT were added and incubated for an additional 48 h.

*** Significantly different (< 0.001) from CPT alone; significance was determined by a Student’s t-test (n = 3). Formation of the DNA-topo I-CPT cleavable complexes was assayed by an SDS-KCl precipitation assay as described in the Methods section. Cells were preincubated with 100 PPNO μM for 6 h before adding CPT for 1 h.

# Fold increase compared to the control values, without CPT.

Effects of ^·^NO on topo I activity was examined by carrying out (a) a cleavage assay with supercoiled p-HOT1 DNA using purified topo I and (b) an SDS-KCl precipitation assay for the formation of a cleavable complex in HT-29 cells in the presence and absence of PPNO and CPT. We examined the effects of various concentrations of PPNO on the DNA cleavage activity of purified topo I in the presence of CPT. Our results show that CPT did not react with ^·^NO/^·^NO-derived species and that topo I remained active in inducing DNA cleavage (Fig 3B). Some degradation of DNA and/or topo I was observed only at high concentrations of PPNO (200 μM or 400 μM ^·^NO, lane 7). These results show that nitrosation of topo I does not significantly modulate the activity of the enzyme as it was fully functional in carrying out DNA cleavage.

This conclusion was further confirmed with an SDS-KCl precipitation assay in HT-29 cells. Data in Table 1 clearly show that similar amounts of topo I-DNA complexes were trapped with SDS-KCl and CPT in the presence or in the absence of PPNO. In the cytokine-induced HT-29 cells, CPT-dependent cleavable complex formation was also similar to that formed in HT-29 cells in the presence or absence of PPNO (Table 1). Taken together, these studies suggest that ^·^NO/^·^NO-derived species do not inactivate topo I protein and the enzymatic function of the nitrosated topo I is fully operative.

In cells containing mutant p53, it has been reported that topo I is closely associated with mutant p53 and that topo I remains hyperactivated [38]. Thus, it is possible that mutant p53 in HT-29 plays a role in the cytotoxicity of CPT in this cell line in spite of the down-regulation of topo I protein. Therefore, to further assess the effects of ^·^NO/^·^NO-derived species on topo I protein in CPT cytotoxicity, we used a wtp53-containing MCF-7 breast cancer cell line.

**Fig 4:**
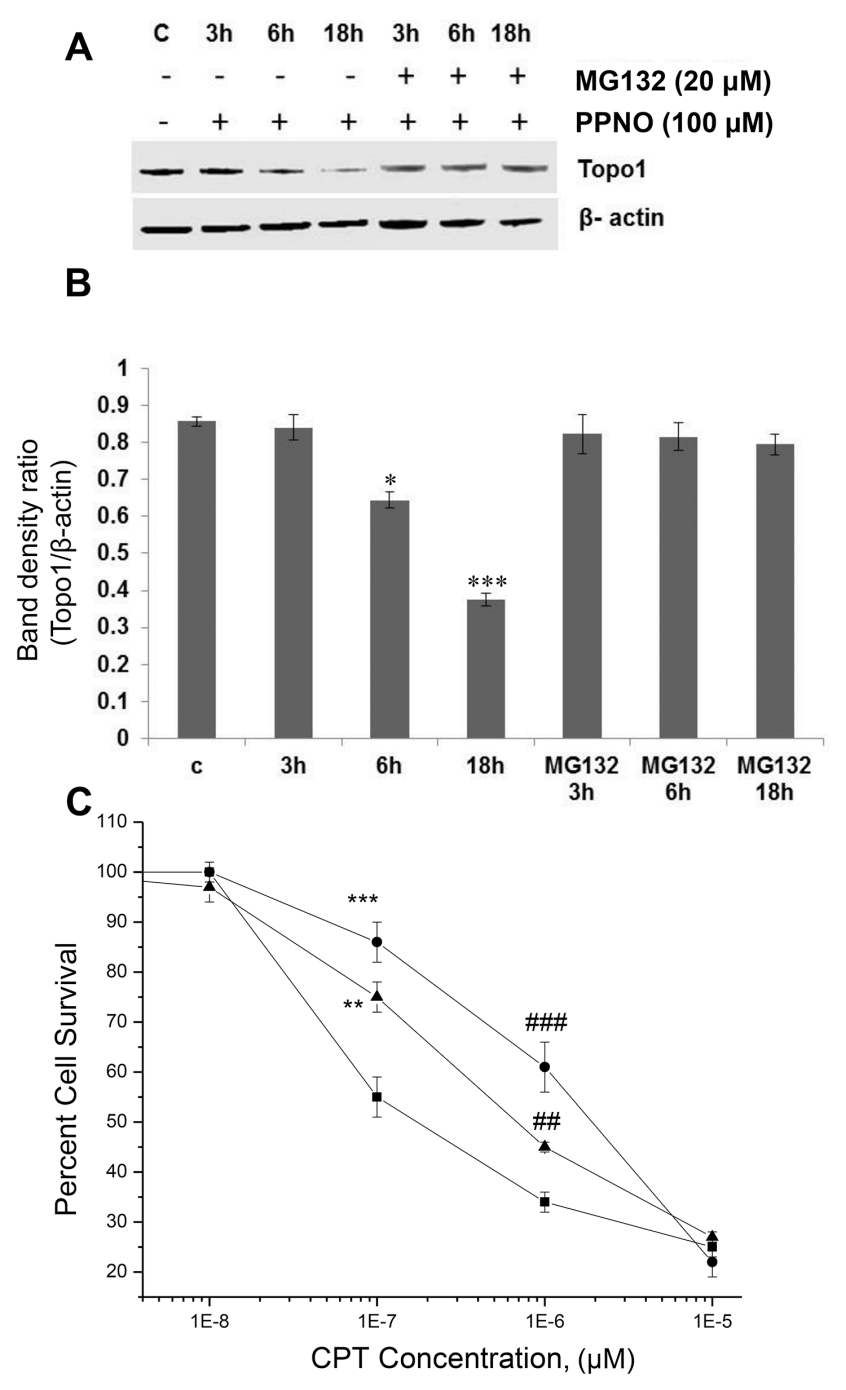
^·^NO-induced down-regulation of topo I protein levels in MCF-7 cells. **(A)** Cells were treated for various times with PPNO and cells were collected, lysed and analyzed by Western blots for topo I, **(B)** Quantification of the resulting topo I protein levels, and **(C)** Cytotoxicity of CPT (■-■) alone and in the presence of PPNO (100 μM; ●-●, 50 μM; ▲-▲) in MCF-7 cells. Values are mean SEM from at least 3 separate experiments carried out in triplicate (n = 3). *P<0.05, **P<0.01, # # and ***P<0.001, # # # and with respect to concentration-matched samples.

Treatment of MCF-7 cells with ^·^NO (via PPNO) resulted in a significant down-regulation of topo I protein levels in a dose- and time-dependent manner (Fig 4). These results are similar to those described for HT-29 cells; however, in contrast to HT-29 cells, the level of topo I protein was further down-regulated up to 18 h following ^·^NO treatment (up to 70% at 100 μM PPNO). ^·^NO did not affect this down-regulation at the topo I gene (Fig 1C). Rather, the down-regulation of the protein was due to degradation of the topo I protein via 26 S proteasome-mediated pathways, as the inclusion of MG 132 completely inhibited this degradation (Fig 4A).

We carried out cytotoxicity studies to evaluate whether this down-regulation of topo I protein would also affect CPT toxicity in MCF-7 cells. Data presented in Fig 4C clearly show that down-regulation by ^·^NO significantly affected CPT cytotoxicity in a dose-dependent manner such that at 100 μM PPNO, MCF-7 cells were significantly (>10-fold) resistant to CPT (Fig 4 and Table 1).

To determine whether this decrease in CPT cytotoxicity in MCF-7 is derived from differential DNA-topo I cleavable complex formation following ^·^NO treatment in the presence of CPT, we examined the formation of the cleavable complexes by the SDS-KCl precipitation assay. Data in Table 1 clearly show that there were no significant differences between the DNA-topo I-CPT cleavable complexes formed in the presence or the absence of ^·^NO treatment, indicating that the difference in CPT cytotoxicity is not due to initial topo I-mediated DNA damage.

^·^NO/^·^NO-derived species have been implicated in the inactivation of caspases and shown to stabilize bcl2 protein, affecting the ability of cells to undergo apoptosis. Therefore, we examined apoptosis in both MCF-7 and HT-29 cells following PPNO treatment in the presence of CPT. MCF-7 cells were found to be extremely resistant to apoptosis in the presence of CPT as observed previously [21] and PPNO treatment had no significant effects on this (S3 Fig). In contrast, while CPT treatment in HT-29 cells induced significant apoptosis, ^·^NO treatment had no further effect (S3 Fig).

**Fig 5:**
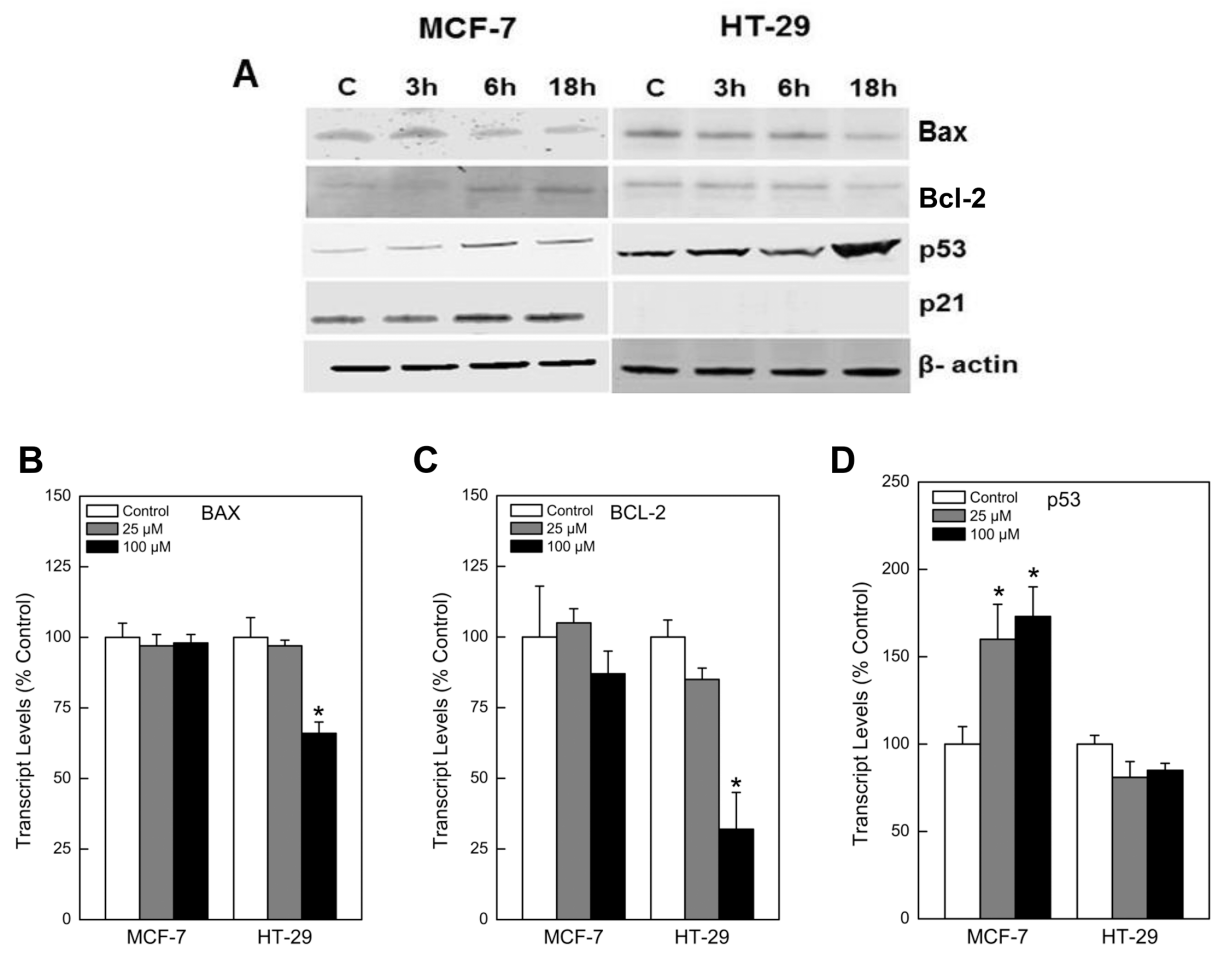
(A) Western blot analysis for Bax, bcl2, p53 and p21 proteins in MCF-7 and HT-29 cells following treatment with PPNO (100 μM) for various times. (B) Transcript levels for Bax, (C) bcl2, and (D) p53 genes following treatment with various concentrations of PPNO. The Western and the RT-PCR analysis were carried out as detailed in the Methods section.

We used Western blots to examine the effect of PPNO on p53, p21, bcl2 and Bax proteins and mRNA levels in both HT-29 and MCF-7 cells. Our results (Fig 5) show that both transcript and proteins levels for wtp53 and p21^WAF/CIP^ were induced (stabilized) by ^·^NO treatment in MCF-7 cells, whereas the mutant p53 and p21 protein levels showed no significant changes in HT-29 cells. Furthermore, the bcl2 protein level was increased (2-3-fold) only in MCF-7 cells while the Bax/bcl2 ratio remained constant in HT-29 cells. This suggests that ^·^NO/^·^NO-derived species modulated (stabilized) bcl2 protein in MCF-7 cells as reported for other cell lines [8, 11].

Because PPNO treatment induced bcl2 protein expression and significantly modulated CPT cytotoxicity only in the wild-type p53-containing MCF-7 cell line, we used pifithrine-α (PFT-α) to evaluate the roles of wtp53 in CPT resistance in MCF-7 cell line. PFT-α is a reversible inhibitor of p53-mediated apoptosis and p53-dependent gene transcription such as cyclin G, p21/waf1, and mdm2 expression [39]. PFT-α has been reported to enhance cell survival after genotoxic stresses such as UV irradiation and treatment with cytotoxic drugs, including doxorubicin, etoposide, and taxol [39]. The protective effect was not observed in p53-null cells expressing a dominant negative mutant of the p53 gene. Additionally, PFT-α has been used in MCF-7 cells to study the role of wtp53 in doxorubicin-induced apoptosis [40].

**Fig 6:**
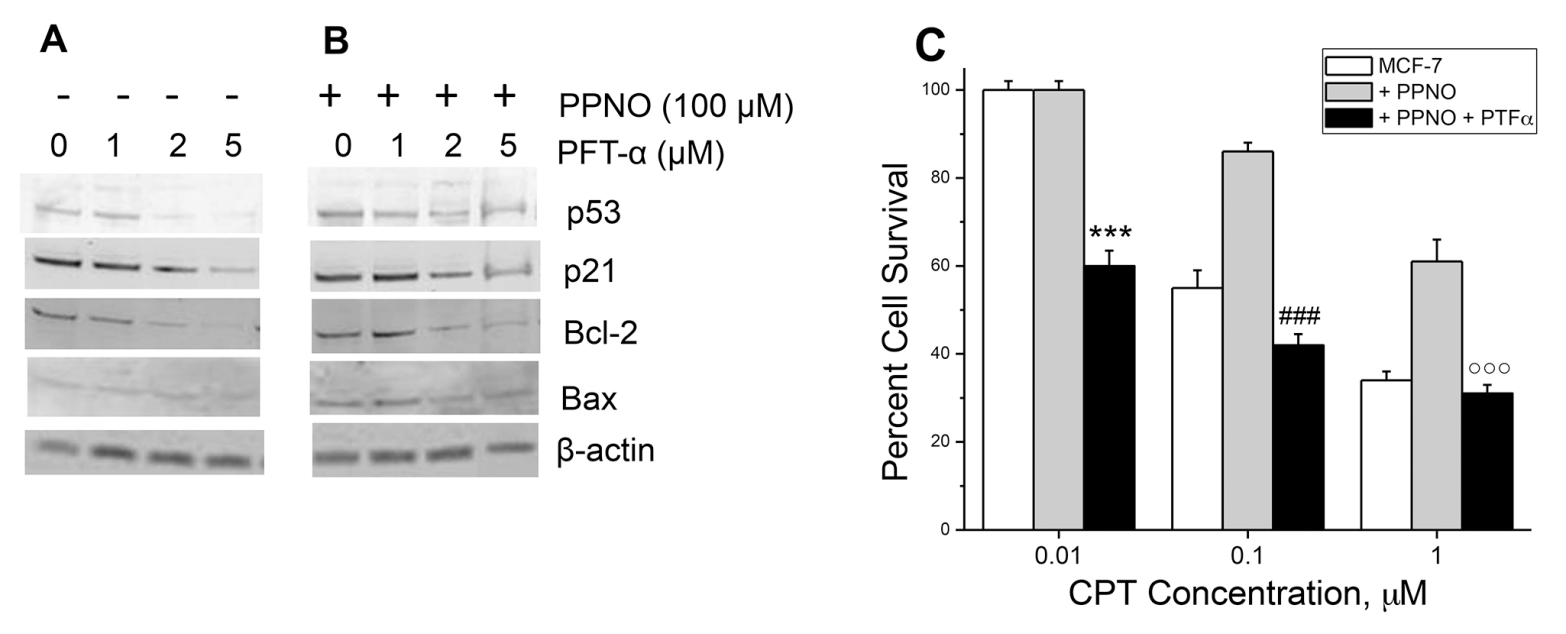
(A) Western blot analysis for p53 and p21, bcl2 and Bax proteins in MCF-7 cells following treatment with different concentrations of PFT-α; (B) in the presence of PPNO (100 μM) for 18h; and (C) cytotoxicity of CPT in the presence of PFT-α (2 μM). The Western analysis and the cytotoxicity studies were carried out as detailed in the Methods section. Values are mean SEM from at least 3 separate experiments carried out in triplicate. Data show mean values ±SEM from three independent (n = 3) experiments. P values <0.001 *** and # # # with respect to both concentration-matched CPT and PPNO treated samples. P values <0.001^○○○^ with respect to concentration-matched PPNO treated samples.

Treatment with PFT-α significantly affected wtp53 protein expression in MCF-7 cells in the presence or absence of PPNO in a dose-dependent manner; PFT-α at 2 μM significantly decreased wtp53 protein expression (Fig 6). Interestingly, PFT-α also inhibited the expression of p21, bcl2 and Bax proteins (Fig 6), suggesting that the induction of bcl2 protein in MCF-7 cells is dependent upon the induction of wtp53 protein expression by PPNO. Therefore, we also used PFT-α to examine CPT cytotoxicity in MCF-7 cells in the presence of PPNO. Data presented in Fig 6B clearly show that PFT-α (2μM) completely reversed CPT resistance induced by PPNO. It is interesting to note that under these treatment conditions, PFT-α also sensitized MCF-7 cells to CPT cytotoxicity (compare Fig 3C to Fig 6C). Taken together, these observations, i.e., inhibition of wtp53 and bcl2 proteins expression by PFT-α and sensitization of MCF-7 cells to CPT by PFT-α clearly demonstrate that ^·^NO/^·^NO-derived species modulated wtp53 functions and bcl2 protein, resulting in the modulation of CPT cytotoxicity in MCF-7 cells.

## Discussion

Studies described here show that topo I protein is nitrosated by ^·^NO/^·^NO-derived species *in vitro*. The human topo I protein contains eight free cysteine groups at positions 300, 341, 386, 453,504, 505, 630 and 733. X-ray crystallography of topo I protein has indicated that the cysteine at 733 is in close proximity to the tyrosine residue at 730 in the active site of the enzyme [15]. Since nitrosation of the protein *in vitro* as well as in tumor cells indicates that the enzyme is active and capable of carrying out DNA cleavage reactions and forming cleavable complexes with DNA in the presence of CPT, it would appear that the cysteine residue at 733 is not nitrosated/or reacted with chemically (PPNO) or intracellularly generated ^·^NO from iNOS during cytokine induction of HT-29 cells. Computer modeling of topo I protein also indicated that the two adjacent cysteines at positions 504 and 505 may not be modified by ^·^NO/^·^NO-derived species due to steric hindrance. However, our mass spectrometric data clearly show that the cysteines at 300, 504, 505 and 630 are observed only as their carbamidomethylated forms and, thus, are most likely modified *in vitro* and in cells.

The significant finding is that topo I protein undergoes down-regulation with time in both HT-29 and MCF-7 cells following treatment with ^·^NO. This down-regulation was not at the topo I gene level, as PPNO treatment did not affect the mRNA of the topo I gene. Instead the down-regulation of topo I protein results from ubiquitin/26S proteasomal-mediated degradation, as a 26S proteasome inhibitor, MG132, abolished ^·^NO-induced topo I down-regulation in both HT-29 and MCF-7 cells.

It has been reported that CPT-induced down-regulation of topo I is involved in the mechanism of CPT cytotoxicity [41, 42]. In addition, one of the mechanisms of resistance has been postulated to involve decreases in target enzymes, e.g., topoisomerase. Our cytotoxicity studies in HT-29 cells suggest that down-regulation of topo I by ^·^NO/^·^NO-derived species is not involved in CPT cytotoxicity. Furthermore, no significant differences were observed in the formation of cleavable complexes in HT-29 cells. While these results are somewhat surprising, sensitivity/resistance to CPT does not correlate well with the cellular levels of topo I or the amounts of cleavage complexes formed in some tumor cells [16, 20, 21].

In contrast, PPNO treatment induced a significant (> 10-fold) resistance in CPT cytotoxicity in MCF-7 cells (Table 1, Fig 4). The kinetics of topo I down-regulation by ^·^NO^·^NO-derived species in MCF-7 cells appeared to be somewhat different from that in the HT-29 cells. While ^·^NO induced a significant (about 50%) down-regulation of topo I protein in HT-29 cells, there was no further down-regulation of the protein after 6 h, whereas in MCF-7 cells protein levels remained significantly (about 70%) depressed even after 18 h. Desai et al. [41, 42] have reported that certain breast cancer cells, e.g., ZR75-1, are resistant to CPT-induced topo I down-regulation and are extremely sensitive to CPT, while BT474 cells, which are sensitive to CPT-induced down-regulation, are resistant to CPT. In this scenario, MCF-7 cells that undergo a rapid and sustained down-regulation of topo I by ^·^NO^·^NO-derived species are significantly more resistant to CPT. Interestingly, down-regulation of the protein had no significant effects on the formation of the DNA-cleavable complexes in the presence of CPT in MCF-7 cells (Table 1). Again, a lack of correlation with DNA-cleavable complex formation and CPT cytotoxicity has been observed before, as other investigators have made similar observations in breast as well as colon cancer cell lines [20, 21].

Desai et al. [41, 42] have suggested that down-stream events from the cleavable complex may be an important determinant of CPT toxicity. While the nature of down-stream events for CPT toxicity is not known, stabilization of wtp53 is important for cell cycle arrest, apoptosis, and repair of DNA damage leading to cell death. Several investigators have shown that inactivation of wtp53 by human papilloma virus (16E6) in MCF-7 cells sensitizes tumor cells to several anticancer drugs, including CPT [22, 43] and cisplatin [44].

^·^NO/^·^NO-derived species have been shown to react/nitrosates bcl2 protein, stabilizing it by inhibiting its proteasomal degradation, resulting in resistance to cisplatin in human melanoma cells [8, 11]. We found that MCF-7 cells are extremely resistant to apoptosis in the presence of CPT compared to HT-29 cells and that the bcl2 protein level was increased (2-3-fold) only in MCF-7 cells while the Bax/bcl2 ratio remained constant in HT-29 cells. This would suggest that ^·^NO/^·^NO-derived species stabilized bcl2 protein in MCF-7 cells, resulting in CPT resistance. Induction and stabilization of bcl2 in MCF-7 cells was dependent upon the induction of wt53 protein, as there was no significant induction of bcl2 protein in the HT-29 cells. Furthermore, when we used PFT-α, which inhibited the induction of wtp53 protein in the presence ^·^NO/^·^NO -derived species, no bcl2 protein was induced in MCF-7 cells. More importantly, under these treatment conditions, PFT-α completely reversed NO-induced resistance of CPT in MCF-7 cells. These events, then, clearly suggest that wt53 is responsible for ^·^NO-induced resistance of CPT in MCF-7 cells by means of stabilizing bcl2 protein.

^·^NO/^·^NO-derived species are known to modulate p53 functions [45–47], inhibit DNA repair proteins [48, 49] and upregulate DNA-PKcs [50], inducing resistance to adriamycin, cisplatin, bleomycin, and x-ray radiation. It is surprising, however, that ^·^NO/^·^NO -derived species-mediated down-regulation of topo I did not contribute to DNA cleavage and CPT resistance in HT-29 cells. It should be noted that we used low concentrations of an ^·^NO-donor (PPNO) with a very short half-life that may not be sufficient to degrade enough of the protein to cause a differential effect on DNA damage. However, during inflammation and in tumor cells infiltrated with macrophages, large amounts of ^·^NO are continuously generated and under these conditions, topoisomerases as well as wtp53 would be under constant attack by ^·^NO. It is, therefore, likely that constant degradation of topo I and up-regulation of p53 and bcl2 proteins would become normal cellular phenomena, causing significant resistance to a wide variety of chemotherapeutic agents. While it is well-documented that the mechanism of CPT cytotoxicity is due to its ability to interact with topo I which induces protein-dependent DNA damage, role (s) of other proteins in the mechanism of CPT cytotoxicity is not clear. Our in-cell nitrosation experiments with PPNO clearly indicated significant increases in nitrosation of a number of proteins, including topo I, over endogenous nitrosation in both MCF-7 and HT-29 cells. Under these conditions of stress resulting in extensive modifications of various proteins by ^·^NO/^·^NO-derived species, it is not unreasonable to assume that other proteins could also play a role in causing CPT cytotoxicity and/or resistance to tumor cells *in vitro* and *in vivo*. In this regard, overexpression of iNOS, a generator of intracellular ^·^NO, is linked to poor prognosis and survival in breast cancer patients [51, 52]. Recently, Heineche et al. [53] have shown that increased production of ^·^NO via elevated NOS2 signaling from tumor microenvironments induces resistance to taxol in certain breast cancer cells. In human breast MCF-7 cancer cells, our studies clearly show that stabilization of bcl2 protein by induction of wt53 protein by ^·^NO/^·^NO-derived species induces significant resistance to CPT, an important clinical drug for treatment of a wide variety of solid tumors. Our results suggest that ^·^NO may be an important modulator of drug resistance in breast cancer patients. Understanding the role of ^·^NO in CPT resistance will provide effective strategies for overcoming drug resistance in cancer patients.

## Supporting Information

S1 Fig Representative LC-ESI-MS/MS spectrum of an unmodified, cysteine containing, TOPO1 tryptic peptide. (PDF)

S2 Fig Representative confocal microscopy for topo I nitrosation in HT-29 and MCF-7 cells following treatment with 100 μM PPNO. (PDF)

S3 Fig Apoptotic Analysis for HT-29 and MCF-7 cells. (PDF)

S1 Table (PDF)
