# Supplementary material for: Nitric Oxide Down-Regulates Topoisomerase I and Induces Camptothecin Resistance in Human Breast MCF-7 Tumor Cells
Source: PLoS One. 2015 Nov 5;10(11):e0141897. doi: 10.1371/journal.pone.0141897 (PMC4635000; doi:10.1371/journal.pone.0141897)
Supplement: S1 Fig — (PDF) [file pone.0141897.s001.pdf]

S1 Fig

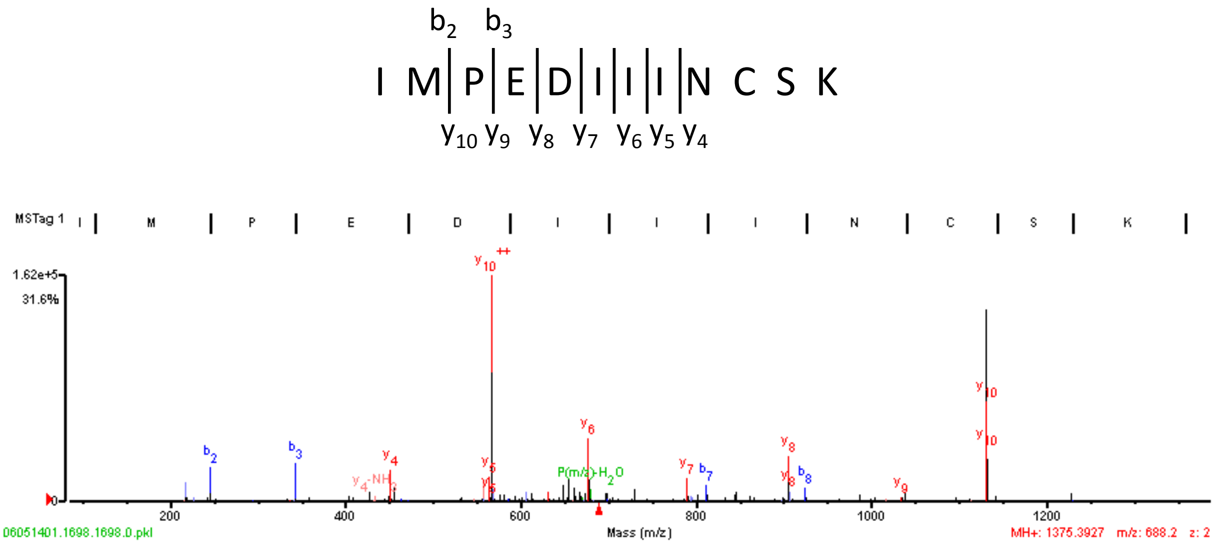

Representative LC-ESI-MS/MS spectrum of an unmodified, cysteine-containing, TOPO1 tryptic peptide. In this instance, an extensive y-ion series allows for the unambiguous assignment of this peptide to tryptic peptide 100 of TOPO1.
