# Supplementary material for: Nitric Oxide Down-Regulates Topoisomerase I and Induces Camptothecin Resistance in Human Breast MCF-7 Tumor Cells
Source: PLoS One. 2015 Nov 5;10(11):e0141897. doi: 10.1371/journal.pone.0141897 (PMC4635000; doi:10.1371/journal.pone.0141897)
Supplement: S2 Fig — (PDF) [file pone.0141897.s002.pdf]

S2 Fig

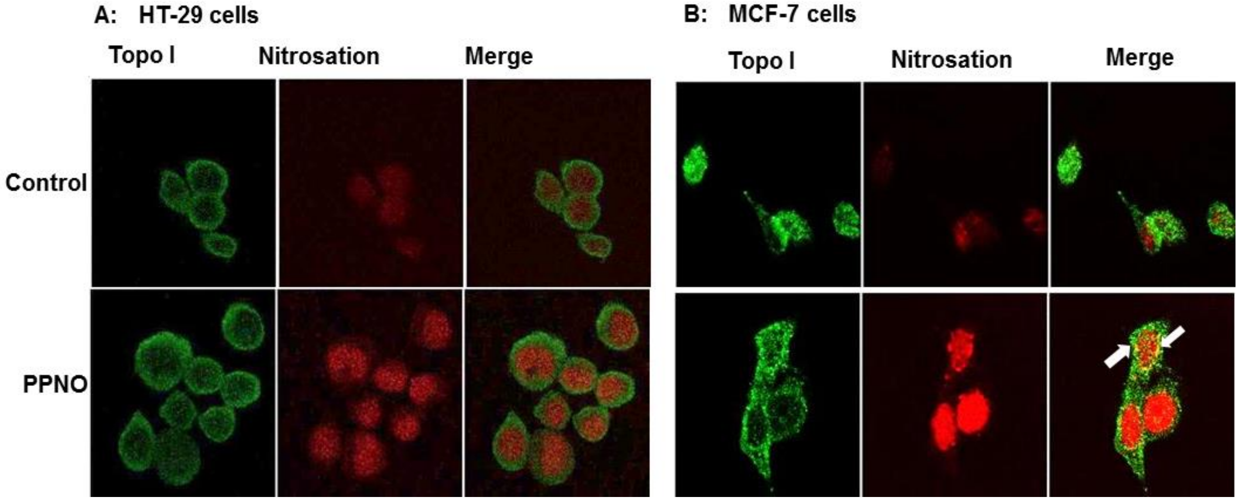

Confocal microscopy: Representative confocal microscopy for topo I nitrosation in HT-29 (Supplementary Figure 2A) and MCF-7 cells (Supplementary Figure 2B) following treatment with 100 μM PPNO.
