# Supplementary material for: Nitric Oxide Down-Regulates Topoisomerase I and Induces Camptothecin Resistance in Human Breast MCF-7 Tumor Cells
Source: PLoS One. 2015 Nov 5;10(11):e0141897. doi: 10.1371/journal.pone.0141897 (PMC4635000; doi:10.1371/journal.pone.0141897)
Supplement: S3 Fig — (PDF) [file pone.0141897.s003.pdf]

## S3 Fig

### Apoptotic analysis for HT-29 and MCF-7 cells

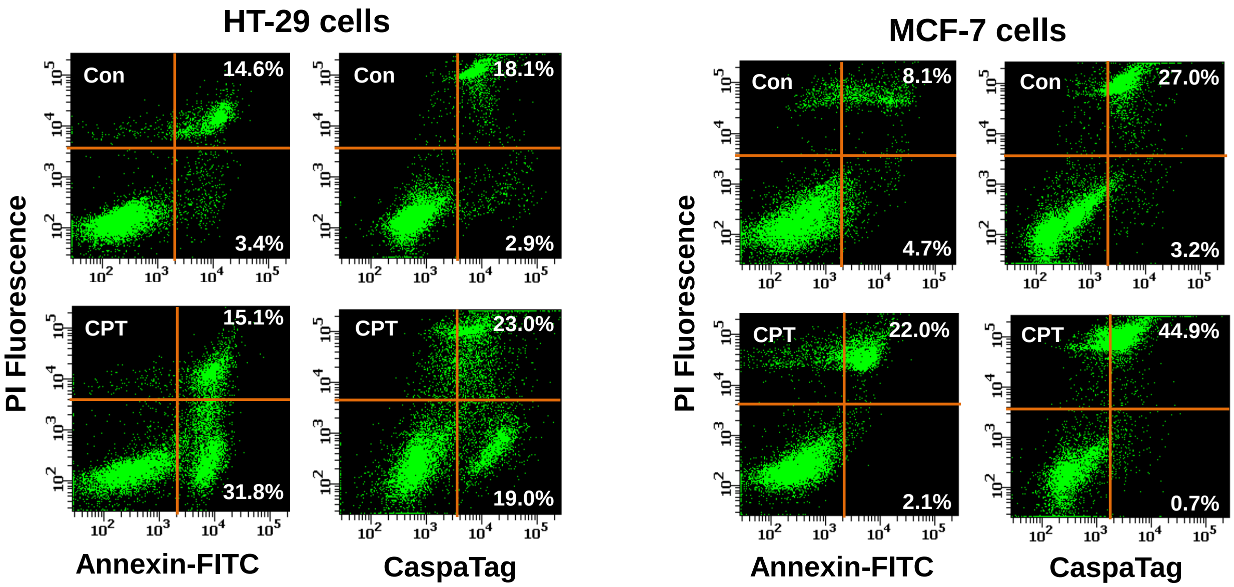


**Apoptotic Analysis for HT-29 and MCF-7 cells**. HT-29 and MCF-7 cells were treated with 10 uM CPT and examined for externalization of phosphatidylserine (Annexin) or caspase activity (CaspaTag). Only the HT-29 cells showed an increase in the Annexin positive / PI negative population of cells characteristic of apoptosis. Furthermore, the HT-29 also showed an increase in the population of cells that were CaspaTag positive / PI negative again characteristic of apoptosis.
