# Supplementary material for: Nitric Oxide Down-Regulates Topoisomerase I and Induces Camptothecin Resistance in Human Breast MCF-7 Tumor Cells
Source: PLoS One. 2015 Nov 5;10(11):e0141897. doi: 10.1371/journal.pone.0141897 (PMC4635000; doi:10.1371/journal.pone.0141897)
Supplement: S1 Table — (PDF) [file pone.0141897.s004.pdf]

S1 Table.

Start Amino Acid	Sequence	Measured (m/z)	Corresponding M+H	Theoretical M+H	Delta mass (Da)
Unmodified TOPO1					
219	(K)FLEHKGPVFAPPYEPLPENVK(F)	603.5	2411	2408.3	2.7
219	(K)FLEHKGPVFAPPYEPLPENVKFYYDGKVMK(L)	591.2	3542.2	3539.8	2.4
249	(K)LSPKAEEVATFFAK(M)	769.7	1538.4	1537.8	0.6
253	(K)AEEVATFFAK(M)	556.7	1112.4	1112.6	-0.2
337	(K)EYGFCIMDNHKER(I)	411.2	1641.8	1641.7	0.1
341	(F)CIMDNHKERIANFKIEPPGL(F) Unmodified (C)	466.1	2326.5	2325.2	1.3
350	(R)IANFKIEPPGLFRGR(G)	573.1	1717.3	1715	2.3
350	(R)IANFKIEPPGLF(R)	673.2	1345.4	1345.8	-0.3
377	(R)IMPEDIIINCSK(D) Unmodified (C)	688.2	1375.1	1375.7	-0.6
377	(R)IMPEDIIINCSKDAK(V) Unmodified (C)	563.8	1689.4	1689.9	-0.5
476	(R)AVALYFIDKLALRAGNEKEEGETADTVGCCSLR(V) Unmodified (C)	591.2	3542.2	3542.8	-0.9
593	(Y)NASITLQQQLKELTAPDENIPAK(I)	841.8	2523.4	2522.4	1
639	(K)TFEKSMMNLQTK(I)	486.6	1457.8	1457.7	0.1
728	(R)ITVAWCKKWGVPIEK(I)	586.9	1758.7	1758	0.7
736	(K)WGVPIEKIYNKTQR(E)	577.9	1731.7	1732	-0.4
736	(K)WGVPIEKIYNK(T)	449.8	1347.4	1346.7	0.6
Carbamidomethylated TOPO1					
131	(K)EDIKPLK(R)	842.509		842.498	0.01
138	(K)RPRDEDDADYKPK(K)	1604.742		1604.766	-0.024
205	(K)WWEEERYPEGIKWK(F)	1935.925		1935.939	-0.014
224	(K)GPVFAPPYEPLPENVK(F)	1753.901		1753.916	-0.015
253	(K)AEEVATFFAK(M)	1112.56		1112.562	-0.003
277	(K)NFFKDWR(K)	1012.501		1012.500	0.001
292	(K)NIITNLSKCDFTQMSQYFK(A) Unmodified (C)	2297.183		2297.094	0.089

300	(K)CDFTQMSQYFKAQTEARK(Q) Carbamidomethyl (C)	2239.115		2239.027	0.088
355	(K)IEPPGLFR(G)	928.525		928.525	0.0001
376	(R)RIMPEDIIINCSK(D) Unmodified (C)	1531.791		1531.797	-0.007
410	(K)VTWLVSWTENIQGSIK(Y)	1860.97		1860.985	-0.016
426	(K)YIMLNPSSR(I)	1080.545		1080.551	-0.006
459	(R)NQYREDWK(S)	1138.526		1138.528	-0.002
489	(R)AGNEKEEGETADTVGCCSLR(V) 2 Carbamidomethyl (C)	2182.93		2182.934	-0.004
509	(R)VEHINLHPELDGQEYVVEFDFLGK(D)	2827.394		2827.394	0
550	(K)NLQLFMENKQPEDDLFDR(L)	2252.06		2252.065	-0.005
559	(K)QPEDDLFDR(L)	1134.502		1134.506	-0.004
559	(K)QPEDDLFDRLNTGILNK(H)	1987.995		1988.008	-0.013
568	(R)LNTGILNKHLQDLMEGLTAK(V)	2225.105		2225.196	-0.091
576	(K)HLQDLMEGLTAK(V)	1355.686		1355.699	-0.013
591	(R)TYNASITLQQQLK(E)	1507.8		1507.812	-0.011
625	(R)AVAILCNHQR(A) Carbamidomethyl (C)	1181.614		1181.621	-0.007
651	(K)LEVQATDREENK(Q)	1431.698		1431.707	-0.009
671	(K)LNYLDPR(I)	890.468		890.473	-0.005
686	(K)WGVPIEK(I)	828.457		828.461	-0.004
